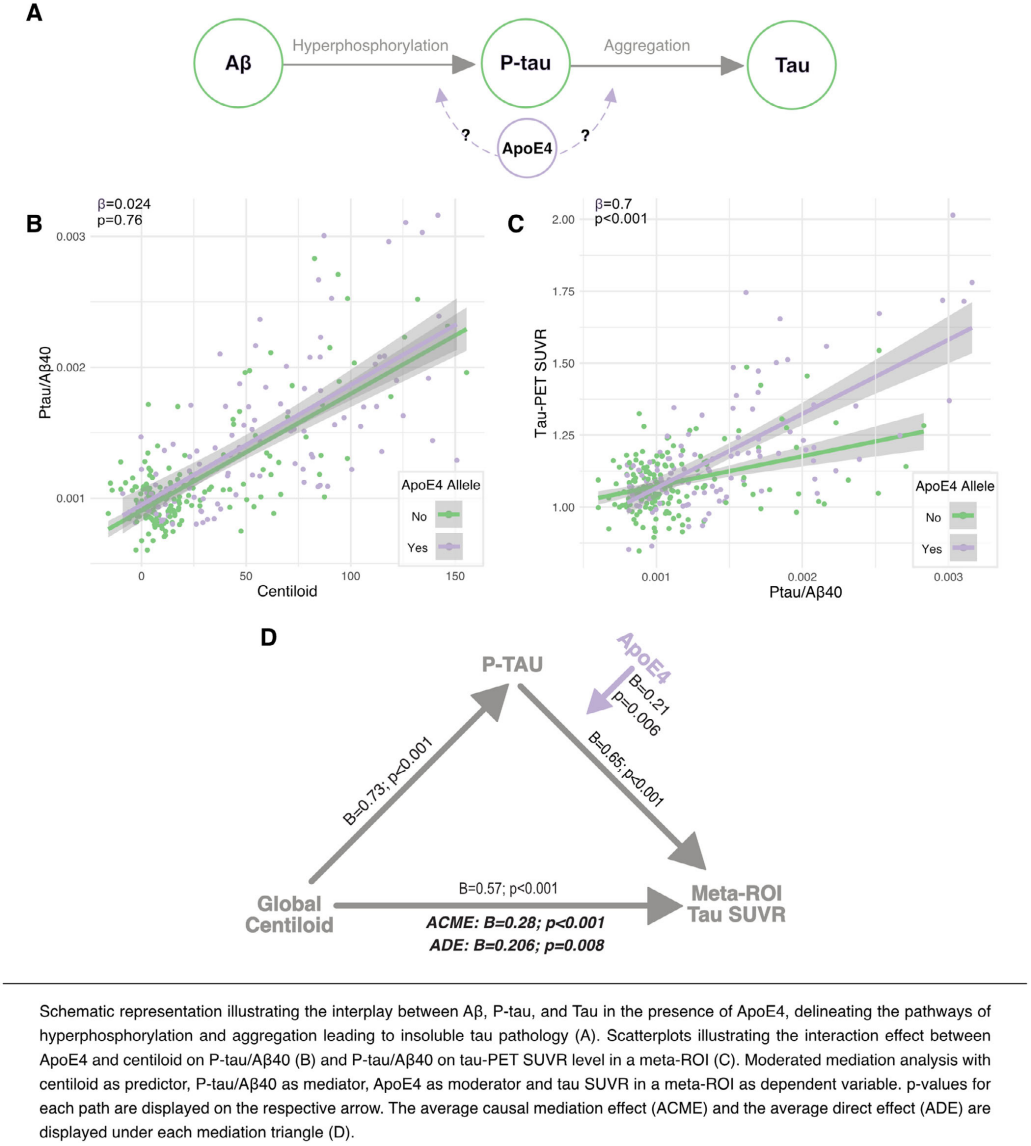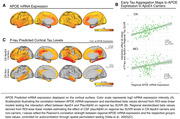# The Role of ApoE4 in The Acceleration of Tau Aggregation

**DOI:** 10.1002/alz.092325

**Published:** 2025-01-09

**Authors:** Anna Steward, Davina Biel, Sebastian Niclas Roemer, Zeyu Zhu, Julia Pescoller, Fabian Wagner, Mattes Gross, Martin Dichgans, Michael Ewers, Amir Dehsarvi, Matthias Brendel, Nicolai Franzmeier

**Affiliations:** ^1^ Institute for Stroke and Dementia Research (ISD), University Hospital, LMU, Munich, Bavaria Germany; ^2^ Institute for Stroke and Dementia Research (ISD), University Hospital, LMU, Munich Germany; ^3^ Department of Neurology, University Hospital, LMU, Munich, Bavaria Germany; ^4^ Institute for Stroke and Dementia Research (ISD), LMU University Hospital, Munich, Munich (Bavaria) Germany; ^5^ German Center for Neurodegenerative Diseases (DZNE), Munich Germany; ^6^ Munich Cluster for Systems Neurology (SyNergy), Munich Germany; ^7^ German Center for Neurodegenerative Diseases (DZNE), Munich, Bavaria Germany; ^8^ Department of Nuclear Medicine, University Hospital, LMU Klinikum, Munich, Bavaria Germany; ^9^ Munich Cluster for Systems Neurology (SyNergy), Munich, Bavaria Germany; ^10^ University of Gothenburg, The Sahlgrenska Academy, Institute of Neuroscience and Physiology, Psychiatry and Neurochemistry, Gothenburg Sweden; ^11^ Institute for Stroke and Dementia Research (ISD), University Hospital, LMU, Munich, Bayern Germany

## Abstract

**Background:**

Understanding modulators of Alzheimer's disease’s (AD) progression is crucial for determining optimal treatment windows and targets. Apolipoprotein E ε4 (ApoE4), i.e. a key AD risk factor, is associated with earlier tau accumulation at lower Aβ levels (Steward et al. 2023), yet, the mechanisms driving this connection remain unclear. Thus, we assessed whether ApoE4 accelerates initial Aβ‐related tau secretion measurable in CSF or subsequent tau aggregation as determined via PET (Figure 1A).

**Method:**

We combined cross‐sectional CSF measures of phosphorylated tau (p‐tau_181_) and Aβ‐PET in 287 APOE genotyped non‐demented (cognitively normal [CN]; mildly cognitively impaired [MCI]) ADNI participants. P‐tau_181_ was adjusted to Aβ40 to account for inter‐individual variability in CSF protein concentrations. Using linear regression, we investigated i) whether ApoE4 was related to accelerated Aβ‐related p‐tau secretion (i.e., Aβ‐PET by ApoE4 interaction on p‐tau_181_), ii) whether ApoE4 accelerated the p‐tau‐induced fibrillisation of tau (i.e. p‐tau_181_ by ApoE4 interaction on tau‐PET) and iii) whether regional effects of ApoE4 on p‐tau‐related tau fibrillisation were associated with ApoE4 mRNA expression levels.

**Result:**

ApoE4 did not moderate the relationship between Aβ‐PET and p‐tau_181_ (Figure 1B, p=0.76) but strengthened the effect of p‐tau_181_ on global Tau‐PET increase (Figure 1C, β=0.7, p<0.001). At the regional level, this ApoE4 x p‐tau_181_ interaction on tau‐PET was correlated with regional APOE mRNA expression in CN (Figure 2B, r=0.5, p_spin_<0.001), but not in MCI (p=0.141), suggesting that ApoE4 drives earliest p‐tau‐induced tau aggregation. Supporting this further, we found that the regional effect of p‐tau_181_ on Tau‐PET was more strongly correlated with APOE mRNA expression in CN ApoE4 carriers (Figure 2C, r=0.54, p_spin_<0.001) than in CN non‐carriers (r=0.4, p_spin_=0.05). Lastly, we confirmed that the effect of Aβ on tau aggregation is mediated by p‐tau increases (Figure 1D, ACME: B=0.28; p<0.001; ADE: B=0.206; p=0.008) which is strengthened in ApoE4 carriers (B=0.21, p=0.006).

**Conclusion:**

ApoE4 promotes p‐tau‐induced tau aggregation, particularly in early disease stages and in regions that express high APOE. This suggests that ApoE4 can trigger earlier Aβ‐related tau spreading most likely due to facilitated p‐tau induced tau aggregation. This suggests that preventing soluble p‐tau increases may attenuate tau aggregation and therefore dementia.